# Genetic identification of marine eels (Anguilliformes: Congroidei) through DNA barcoding from Kasimedu fishing harbour

**DOI:** 10.1080/23802359.2021.1996291

**Published:** 2021-11-11

**Authors:** Ranjana Bhaskar, Mrinal Kumar Das, E. Agnita Sharon, Rupavath Rajendar Kumar, Chandika R. G.

**Affiliations:** a Zoological Survey of India, Southern Regional Centre, Chennai, India; b Zoological Survey of India, Marine Biology Regional Centre, Chennai, India

**Keywords:** Muraenesocidae, COI, phylogeography, ophichthidae, congroidae, barcode

## Abstract

Along with the mysteries of their body's shape like snakes, marine eels have fascinated biologists for centuries. Information on the molecular taxonomy of marine eels is scarce from the Southeast Indian region and hence, the present study aimed to barcode marine eels collected from Kasimedu fishing harbor, Chennai, Tamil Nadu. A total of 44 specimens were collected and DNA barcoding was done with a COI marker. The evolutionary history was inferred using the BA method. We observed 17 species, 10 genera, 4 families from the suborder Congroidei of which the genus Ariosoma and Conger were found to be predominant. The species of the family Muraenesocidae and Congridae are highly variable. The average Kimura two-parameter (K2P) distances within species, genera, and families were 3.08%, 6.80%, 13.80%, respectively. Maximum genetic distance (0.307) was observed between the species *Muraenesox cinereus* and *Ariosoma sp.*1. BA tree topology revealed distinct clusters in concurrence with the taxonomic status of the species. A deeper split was observed in *Uroconger lepturus*. We sequenced for the first-time barcode of *Sauromuraenesox vorax* and a new species *Ophichthus chennaiensis* is the gap-filling in identifying this taxon in the Indian context. We found a correct match between morphological and genetic identification of the species analyzed, depending on the cluster analysis performed (BINs and ASAP). This demonstrates that the COI gene sequence is suitable for phylogenetic analysis and species identification.

## Introduction

Congroidei is the most ecologically diverse Suborder in the order Anguilliformes comprising 5 families, 97 genera, and 498 species (McCosker 2010). Anguilliformes can be identified using body proportions, presence or absence of fins, the position of fin origins, nature of nostrils and gill opening, and the number of vertebrae. Many external features in Anguilliformes are reduced or converged making them very similar to each other morphologically, thereby requiring high-level expertise to distinguish one species from the other based on morphometric analysis (Muchlisin et al. 2017). Furthermore, the life history of eels from being a pelagic leptocephalus larva followed by metamorphosis to mature individuals before spawning makes eels difficult to identify due to the lack of metamorphic stages recorded for each species. From an evolutionary perspective, a morphological examination may not always help in distinguishing species since the causation of difference is not always independent and reproductively isolated taxa can be morphologically indistinguishable. Bearing in mind these characteristics of this taxa, we emphasize that there are numerous unrecorded species, their metamorphic stages and poorly studied the taxonomy of eels from the Indian coasts.

This situation about documenting eel species found in Indian waters can be addressed using DNA barcoding. The mitochondrial genome which has features like high copy number, maternal inheritance, lack of introns, limited recombination and varying degrees of mutations in different regions (Saccone et al. 1999) is apt for studying inter-and intraspecific differences of various taxa. Specifically, the COI gene has a greater phylogenetic signal than others in the mitogenome arising because of high nucleotide substitution in the third position of a codon triplet (Knowlton and Weigt 1998) thereby aiding in identifying species. Sanger sequencing, being the gold standard in sequencing technology gives accurate data to ascertain the species present in a particular area. Classical barcoding using Sanger sequencing, metabarcoding, and eDNA using next-generation sequencing could help in advances in the field of biodiversity research. These techniques can be of relevance in terms of species such as eels that have such a long and complex catadromous life cycle, where the tracking of individuals through the many life stages is almost impossible (Hanzen et al. 2020). The limitations of morphology-based identification systems and the diminishing group of taxonomists lead to the implementation of a molecular approach for species identification.

Documentation of species available in a region is the primary step before management or conservation strategies can be framed. Barcode data helps in improving biodiversity surveys by contributing toward monitoring eel distribution and conservation (Hanzen et al. 2020). In this study, mitochondrial COI marker will be used for identifying species of suborder Congroidei which is a distinguishing region for species identification and systematic research on aquatic fauna in India (Kundu et al. 2019). This is a robust tool for examining intraspecies, interspecies variations, and the deeper splits within species (Kvie et al. 2012). With a large gap in identifying this taxon in the Indian context, we made an exploratory effort to identify species belonging to sub-order Congroidei from the Southeast coast of India using a DNA barcode.

## Materials and methods

### Sample area

**Figure 1.**
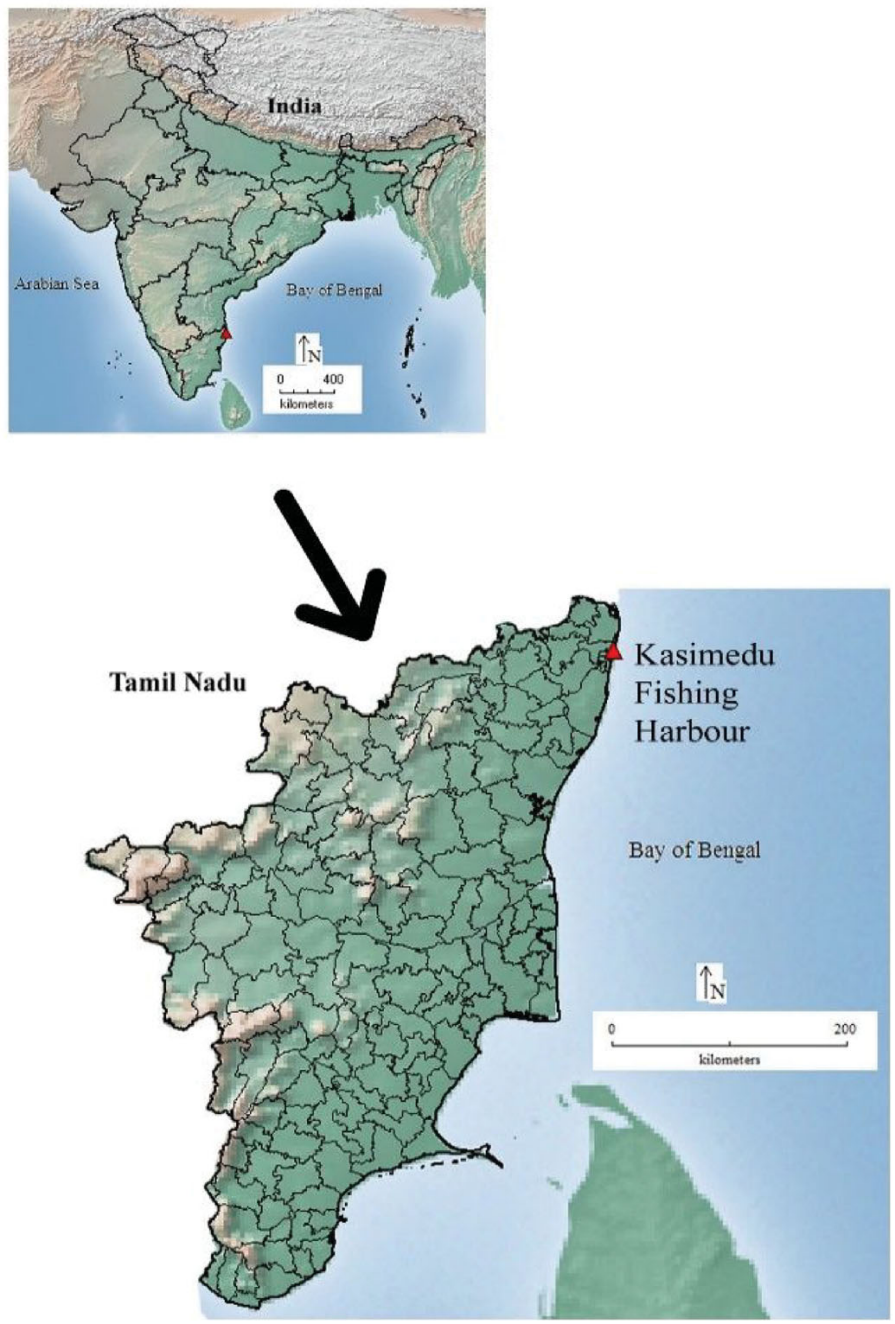
Collection of the sampling locality for the specimens collected in this study.

Kasimedu or Royapuramarea (Figure 1) fishing harbor is the major fishing landing center in Chennai (13°7′29.40″N, 80°17'46.40" E), Tamil Nadu. Trawler and bigger boats remain in the sea for over a week while fishes are caught and segregated based on their appearance (roughly species-wise). Eels also appear in these catches and are directly linked with the human as a food source with high commercial value (Sarkar 2018). Forty-four specimens were collected opportunistically from the Kasimedu landing center. To check the authentication of the species, we downloaded the sequences from the NCBI database and included them in the study.

### DNA extraction and PCR amplification

Forty-four specimens were first morphologically examined by the experts in-house at the Zoological Survey of India up to the genus or species level followed the identification keys of Carpenter & Niem (1999). After identification, 1 sq cm of lateral muscle was excised and stored at −20 °C until DNA isolation. DNA was isolated using a modified procedure of the phenol-chloroform method (Ruzzante et al. 1996). The quality of the extracted DNA was checked in 0.8% agarose in a horizontal electrophoresis system. Primer pair Fish F1 (5-TCAACCAACCACAAAGACATTGGCAC-3′) and Fish R1 (5-TAGACTTCTGGGTGGCCAAAGAATCA-3′) were used for amplifying COI fragments (Ward et al. 2005). We used thermal cycler GX200 (Eppendorf) for PCR. PCR reaction was amplified in a final volume of 25 µl and contained 1.5 units of Taq DNA polymerase, 1X PCR buffer, 1.5 mM MgCl_2_, 0.2 mM of each dNTP, 5 pmol of each primer, and 2 µl (25–50ng) of genomic DNA. Amplification condition was performed with the initial denaturation at 94 °C for 5 min followed by 35 cycles at 94 °C for 30 s, 50 °C for 30 s and 72 °C for 1 min, with a final extension of 72 °C for 10 min. PCR products were purified with a Qiagen PCR purification kit. PCR products that yielded a clear band on agarose gel electrophoresis were sequenced bidirectionally with BigDye Terminator chemistry on an ABI 377 Genetic Analyzer.

### Data analysis

Sequences obtained were checked and edited manually for miscalling and base spacing using BioEdit V7.0 (Hall 1999). The alignment was done in CLUSTALW (Thomson 1997). Each sequence was confirmed through BLASTn (Basic Local Alignment Search Tool) (https://blast.ncbi.nlm.nih.gov). The intraspecific, interspecific, and intergeneric genetic divergences were assessed by the Kimura-2-parameter (K2P) model in MEGAX (Kumar et al. 2018). Species clustering assessed the correspondence between species identification and DNA barcodes by the automatically Barcode Index Number (BIN) clustering (Ratnasingham and Hebert 2013) and Assemble Species by Automatic Partitioning (ASAP) analysis (Puillandre et al. 2021) was performed using default parameters using the web interface (https://bioinfo.mnhn.fr/abi/public/asap/asapweb.html). We also constructed a phylogenetic tree using MrBayes 3.2 (Ronquist et al. 2012) by selecting nst = 6 for GTR + G + I model test with four (one cold and three hot) metropolis-coupled MCMC algorithm and run for 1,000,000 generations with 25% burn-in with trees saving at every 100 generations (Ronquist and Huelsenbeck 2003). The phylogenetic tree was edited in FigTree 1.4.2 (Rambaut 2012).

## Results

Barcode sequences (COI) were generated from 44 muscle samples collected from Kasimedu Landing Center and deposited in the GenBank database and BOLD Systems^®^database (Ratnasingham and Hebert 2007) (Table 1). Eels were first morphologically identified and then identified by DNA barcoding. In cases where morphological specimens were also not consistent, the specimens were identified to the family or genus level with the help of NCBI and BOLD databases. DNA barcoding of 11 specimens did not identify the species level due to the lack of available sequences in NCBI databases. To see the clustering pattern and authentication of species identification, we have downloaded all the haplotypes of the respective studied species and genera from NCBI. Out of 44 studied specimens, 12 species and 11 specimens at the genera level were identified. So a total of 17 species was identified as belonging to 10 genera and 4 families in the suborder Congroidei. Out of 10 genera, 3 were identified with the following genus: *Ariosma*, *Gnathophis* and *Facciolella*. Seven sequences of the genus *Ariosoma* and two sequences of the genus *Gnathophis* were not similar when multiple sequence alignment was done and a BLAST search did not yield species name. Therefore, the sequence clusters were labeled as *Ariasoma sp.* 1; *Ariosoma sp.* 2; *Gnathophis sp.*1*; Gnathophis sp.*2. The species *Sauromuraenesox vorax* was sequenced for the first time that represented new additions to the BOLD and NCBI database. COI gene of *Ophichthus chennaiensis*, (Das et al. 2020), a new species is also included in this study and submitted in both databases.

**Table 1.** List of species included in the study for COI sequence analysis and accession number and barcode index numbers from suborder Congroidei.

Family	Genus	Species	No. of samples	GenBank ID	BINs
Congridae	Ariosoma	Ariosoma meeki	2	MW311323- MW311324	BOLD:AAF6666
		Ariosoma shiroanago	1	MW311321	BOLD:ADC9223
		Ariosoma sp. 1	4	MW311322, MW311325- MW311327	BOLD:AEI5786
		Ariosoma sp. 2	3	MW311318- MW311320	BOLD:AEI5787
	Conger	Conger cinereus	3	MW306768- MW306770	BOLD:AAD1242
	Gnathophis	Gnathophis musteliceps	2	MW306762- MW306763	BOLD:AEJ2482
		Gnathophis sp. 1	1	MW306760	BOLD:AAG4489
		Gnathophis sp. 2	1	MW306761	BOLD:ABA5210
	Uroconger	Uroconger lepturus	10	MW351781- MW351785, MW310970- MW310973, MW387622	BOLD:AAD1243
					BOLD:AAJ8378
Muraenesocidae	Gavialiceps	Gavialiceps taiwanensis	3	MW351778- MW351780	BOLD:AEJ8964
					BOLD:AAF4895
	Muraenesox	Muraenesox bagio	1	MW306767	BOLD:ACK7558
		Muraenesox cinereus	3	MW306764- MW306766	BOLD:ACB5037
	Sauromuraenesox	Sauromuraenesox vorax	5	MW044564- MW044568	BOLD:AEI3835
Nettastomatidae	Facciolella	Facciolella oxyrhyncha	1	MW306758	BOLD:AAB6640
		Facciolella sp.	2	MW306756- MW306757	BOLD:AEJ3046
Ophichthidae	Ophichthus	Ophichthus chennaiensis	1	MW366902	BOLD:AEI1684
	Pisodonophis	Pisodonophis cancrivorus	1	MW306759	BOLD:AAW5582

After alignment, a 650 bp sequence was obtained. The average transitional pairs (si = 69) were more frequent than the average transversional pairs (sv = 50) with an average ratio (R = si/sv) of 1.38. The intraspecific, interspecific and intergeneric genetic divergences were analyzed (Table 2) with NCBI haplotypes. For the genetic variation, we included only the sequences of the present study species haplotype downloaded from NCBI to see the intraspecies variation. Genetic distance within species ranged from 0 to 13.10%. The genetic distances between genus ranged from 0.82to 14.38%, with an average of 6.43%. The pairwise genetic distance was observed between the species (Table 3). Kimura 2-parameter (K2P) genetic distance was highest (0.307) between the species *Muraenesox cinereus* and *Ariosoma sp.*1. The lowest genetic distance (0.1019) was between the species *Ariosoma shiroanago* and *Ariosomasp.*1 (Table 3).

**Table 2.** Genetic distance (% K2P) was observed within various taxonomic levels with NCBI data.

Comparison within	Taxa	Mean	Minimum	Maximum	Standard error	
Species	17	3.08	0.00	13.10	0.14	
Genus	10	6.80	0.82	14.38	0.67	
Family	4	13.80	6.03	20.83	0.38	

**Table 3.** Pair-wise genetic distance using the kimura 2-parameter model (K2P) between 17 species of marine eels from suborder Congroidei with NCBI haplotype.

	1	2	3	4	5	6	7	8	9	10	11	12	13	14	15	16
1 Urocongerlepturus																
2 Sauromuraenesoxvorax	0.2137															
3 Gnathophis sp.1	0.2157	0.2400														
4 Gnathophis sp. 2	0.2194	0.2425	0.1161													
5 Gnathophismusteliceps	0.2360	0.2339	0.2024	0.1737												
6 Gavialicepstaiwanensis	0.2143	0.2304	0.1951	0.2034	0.2122											
7 Facciolella sp.	0.2132	0.2161	0.2309	0.2212	0.2361	0.2036										
8 Facciolellaoxyrhyncha	0.2204	0.1960	0.2223	0.2031	0.2290	0.2215	0.1466									
9 Ophichthuschennaiensis	0.2094	0.1902	0.1980	0.1958	0.2219	0.2223	0.2185	0.2017								
10 Pisodonophiscancrivorus	0.2176	0.2149	0.2186	0.2038	0.2239	0.2079	0.2111	0.2036	0.1712							
11 Ariosoma sp.1	0.2576	0.2573	0.2805	0.2771	0.2724	0.2779	0.2576	0.2657	0.2376	0.2687						
12 Ariosomashiroanago	0.2324	0.2439	0.2503	0.2562	0.2670	0.2581	0.2211	0.2316	0.2130	0.2496	**0.1019**					
13 Ariosomameeki	0.2452	0.2663	0.2713	0.2602	0.2676	0.2752	0.2513	0.2533	0.2415	0.2627	0.1685	0.1449				
14 Ariosoma sp. 2	0.2378	0.2323	0.2654	0.2485	0.2605	0.2577	0.2257	0.2376	0.2082	0.2438	0.1600	0.1572	0.1892			
15 Muraenesox cinereus	0.2407	0.2468	0.2174	0.2225	0.2574	0.2395	0.2701	0.2381	0.2251	0.2343	**0.3070**	0.2834	0.2728	0.2732		
16 Muraenesoxbagio	0.2322	0.2199	0.2288	0.2244	0.2559	0.2334	0.2487	0.2297	0.2076	0.2286	0.2847	0.2679	0.2853	0.2673	0.1332	
17 Conger cinereus	0.2218	0.2082	0.2112	0.2250	0.2645	0.2402	0.2446	0.2280	0.1998	0.2389	0.2901	0.2614	0.2664	0.2455	0.2623	0.2436

**Figure 2.**
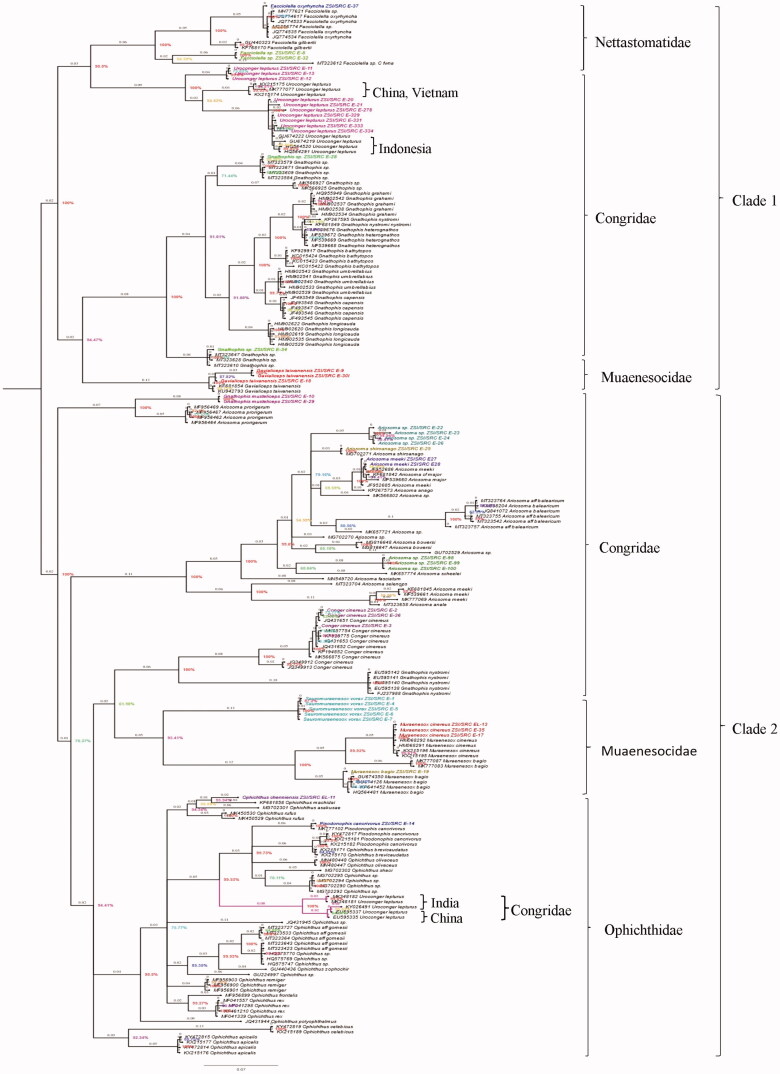
The Bayesian Inference (BI) analysis shows the multiple clades in marine eels of COI gene with NCBI database. Color specimens show the present study. Color branches indicate the separate group of *U. lepturus*. Values near branches show Bayesian posterior probability (PP).

**Figure 3.**
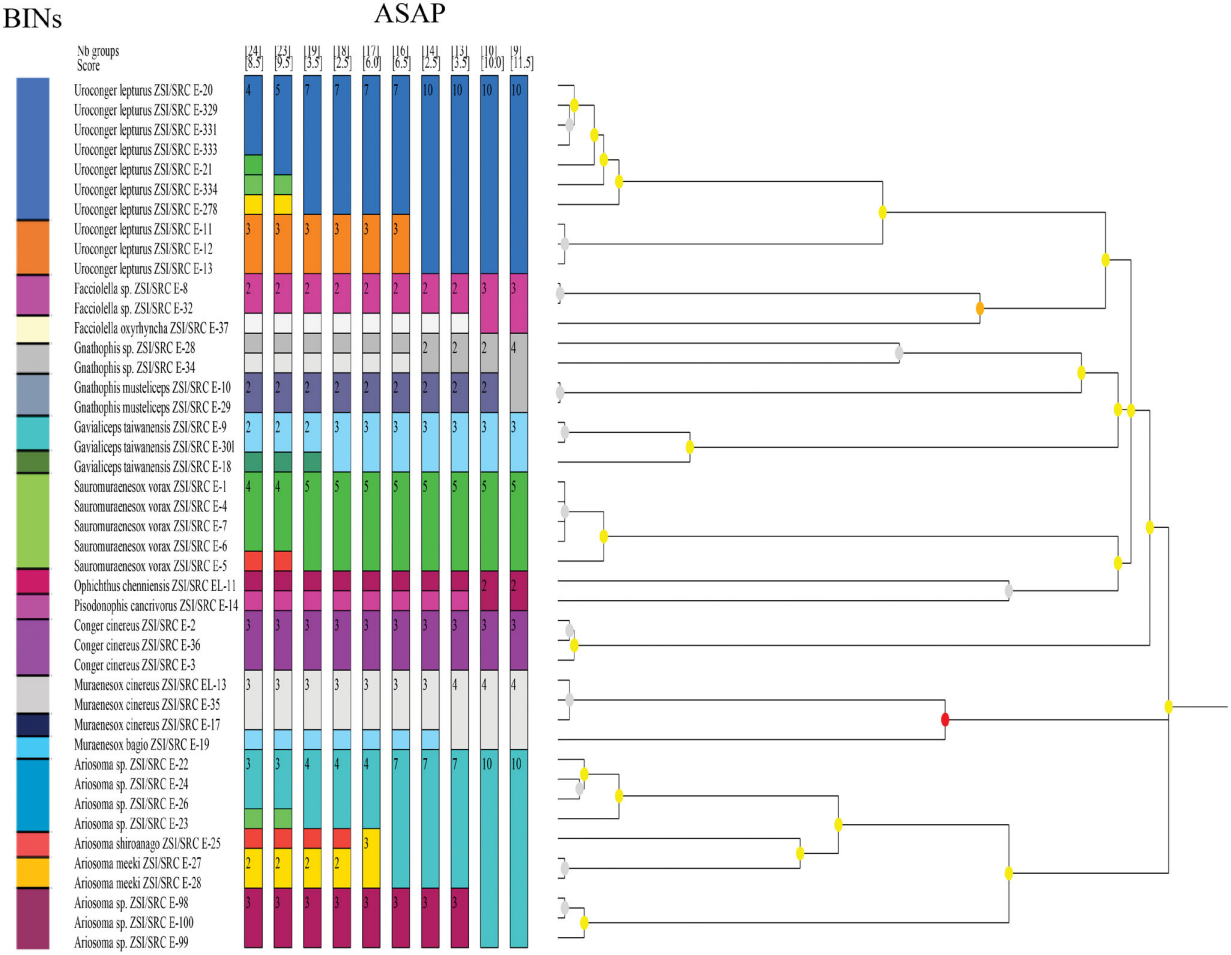
COI gene species delimitation of suborder Congroidei by ASAP and BINs. Colors represent unique partitions.

The topology patterns in the Bayesian method were examined with the NCBI dataset (Figure 2). The Bayesian Inference (BI) tree showed two major clades, the first clade clustered the families Nettastomatidae, Congridae, and Muraenesocidae. The second clade clustered with Ophichthidae, Congridae, and Muraenesocidae. The families Congridae and Muraenesocidae were found in both the clades, which means they are a highly versatile family. All species are separated with high bootstrap values. Specimen of the same species and genus clustered together. *Sauromuraensox vorax* clustered with *Muraenesox cinereus* and *Muraenesox bagio*. The genus *Gnathophis* and *Gavialiceps* were observed as a monophyletic clade. The species *Gnathophis musteliceps* was found clustered with *Ariosoma prorigerum* in clade 2 (Figure 2). Genus *Pisodonophis* and *Ophichthus*, which belongs to the family Ophichthidae, clustered together. A deeper split was observed in the species of *Uroconger lepturus* (Figure 2). The studied ten samples of *Uroconger lepturus* with a branch length of 0.04 and 0.06 formed separate groups. This looks unusually higher than other defined species in our dataset, indicating cryptic diversity of *Uroconger lepturus*. Figure 2 showed four groups of *Uroconger lepturus* with NCBI data. One group of *Uroconger lepturus* (NCBI data) was clustered with *Ophichthus sp*. Species delimitation analyses of the present study are summarized in Figure 3. Assemble Species by Automatic Partitioning (ASAP) analysis observed the 10 best partitions with species delimitation (Figure 3). ASAP observed 24 MOTUs and 19 BINs assigned by BOLD for the sequences generated in this study (Table 1, Figure 3). *Muraenesox bagio* and *Muraenesox cinereus* shared the same BIN (BOLD:ACB5037) (Table 1). *Uroconger lepturus* was divided into two BINs (BOLD:AAD1243 and BOLD:AAJ8378) and *Gavialiceps taiwanensis* into two BINs (BOLD:AFJ8964 and BOLD:AAF4895) (Table 1).

## Discussion

Morphological traits highly overlap among some species of eels making species identification difficult using external traits alone, thus leading to a molecular approach for species identification (Mehta 2009; Ji et al. 2012). The effectiveness of DNA barcodes for species identifications, including marine specimens, has been massively documented in the last two decades (Lakra et al. 2016). DNA barcode from the COI region was used to identify species belonging to the sub-order Congroidei. In this study, nine specimens were unable to be identified until the species stage. Specimens that were unidentified until species using traditional morphometric observation did not yield a species result in NCBI-BLAST. These belonged to the genus *Ariosoma*, and *Gnathophis* which might be undescribed species. A total of 17 species were identified which belonged to 10 genera and 4 families in the suborder Congroidei.

Intraspecific divergence was observed ranging from 0 to 13.10% with a mean of 3.08% which is much higher than previously reported for fishes (Ward et al. 2008; Pereira et al. 2013), where the majority of intraspecific divergence was between 1 and 2% in many fishes (De Brito et al. 2015). This may be increasing in depth; there were increases in the dispersion and nearest neighbor distances among species within the trait space (Bowler and Benton 2009; Clobert et al. 2009). Genetic divergence was ranging from 6.03 to 20.6% within the family (Table 2). The genetic distance (K2P) was larger at higher taxonomic levels, and the increases in genetic distances (K2P) above the species were able to differentiate genera, families, orders, characterized by the constant increase in genetic variation (Hubert et al. 2008).

In this study, the Bayesian Inference (BI) tree was consistent with the morphological identification at the species level, demonstrating the examined species could be authenticated by the barcode approach. To authenticate the unknown species at the genera level of the present study, we downloaded all the haplotypes from NCBI from genera and species level to check the cluster pattern. Bayesian Inference (BI) tree analysis confirms the taxonomic status of studied species. Deeper divergence (12.8% and 13.1%) was observed from the samples of *Uroconger lepturus* and *Ariosoma meeki*, showing much intraspecies differentiation with NCBI data. This divergence may be due to the higher variability of the COI gene sequence at the genus level. The10 barcodes of *Uroconger lepturus* in the present study, after a branch length of 0.9, divide into two clusters with a branch length of 0.04 and 0.06. Four separate clusters were observed in *U. lepturus*, two in studied species and two in NCBI data. One group of *U. lepturus* of the present study clustered with China and Vietnam, and the Second group with Indonesia (Figure 2). One group of *U. lepturus* of NCBI data was grouped with genus *Ophichthus*. The cryptic diversity indicated by *Uroconger lepturus* and *Ariosoma meeki* is a common occurrence in marine species (Ma et al. 2008; Hubert et al. 2012) (Figure 2). This phenomenon can be attributed to the wide distribution of this species arising chiefly because of the pelagic larval stage. The *Uroconger lepturus*, a single species of the genus *Uroconger* is widely distributed from the western Indian Ocean to the western Pacific, as far north as Japan (Smith 1989a), and it appears to be an abundant species of marine eel in the western Indian Ocean (Amir et al. 2005). When we look within the tree intragenerically, *Ariosoma meeki* and *Ariosoma shiroanago* were claded together like sister clades. In Figure 2, *Ariosoma meeki* shows that most diverse groups formed two separate clades.

*Sauromuraesox vorax* showed more genetic relatedness to species from the genus *Muraenesox*. Data confirmed that *S. vorax* clustered with the *M. cinereus* and *M. bagio* as all three species are from the same family under Muraenesocidae (McCosker John et al. 1998). The barcode of a new species *Ophichthus chennaiensis* confirmed to cluster under the family Ophichthidae. Genus *Uroconger* comes under the family Congridae but it is clustered with clade under the family Nettastomatidae. Family Congridae which has over 180 documented species has been recorded as polyphyletic in previous studies (Tang and Fielitz 2013), possibly because of sequence divergence that shared many polymorphic sites in the ancestral species (Austerlitz et al. 2009). The BA tree revealed an identical phylogenetic relationship among the species. The phylogenetic relationship among the species and genera was recognized, and similar species were grouped under the same nodes while dissimilar species were clustered under separate nodes. The nodes were supported by high bootstrap values (90–100%). Congeneric species always clustered together and, in most cases, so did the confamilial species. Species clustering and delimitation were assessed by the ASAP analysis and BINs (Figure 3). As a result of these analyses, 24 MOTUs are observed by ASAP and 19 BINs created by BOLD. Cryptic diversity was observed in the species *Uroconger lepturus* and *Gavialiceps taiwanensis*. Delimitation efficiency is influenced by the number of haplotypes per species, the geographic distance between sampling points of individuals of the same species (Magoga et al. 2021).

The result of the present study contributes a large gap in identifying this taxon in the Indian context, this made an exploratory effort to identify species belonging to sub-order Congroidei from the Chennai Southeast coast of India using DNA barcode. A deeper split in the *Uroconger lepturus* barcodes in the present study confirmed the taxonomic position. Species *Ophichthus chennaiensis* and *Sauromuraesox vorax* is the new addition in NCBI and BOLD database. The success of using barcoding for species identification strongly depends on the presence of reference sequences available in public databases and the existence of specimen vouchers correctly identified. These validate the match between DNA barcode clusters and morphological identification (Peninal et al. 2017). In the present study, the phylogenetic tree showed maximum genetic relatedness with the sequenced results. Therefore, our findings demonstrate that the COI gene sequence is suitable for phylogenetic analysis and species identification.

## Data Availability

The data that support the findings of this study are original and openly available in NCBI at https://www.ncbi.nlm.nih.gov/. Accession number: MW311323- MW311324, MW311321, MW311322, MW311325- MW311327, MW311318-MW311320, MW306768-MW306770, MW306762-MW306763, MW306760, MW306761, MW351781-MW351785, MW310970-MW310973, MW387622, MW351778-MW351780, MW306767, MW306764-MW306766, MW044564-MW044568, MW306758, MW306756-MW306757, MW366902, MW306759
